# Naïve Garter Snake Chemosensory Preferences Corroborate Diet of Wild Individuals and Reveal Behavioral Convergence for Foraging in Deep Water

**DOI:** 10.1093/iob/obag030

**Published:** 2026-06-18

**Authors:** E Carrillo, M Berry, D S Rojas, I A Somarriba, R S Mehta

**Affiliations:** Department of Ecology and Evolutionary Biology, University of California at Santa Cruz, Santa Cruz, CA 95064 USA; Department of Ecology and Evolutionary Biology, University of California at Santa Cruz, Santa Cruz, CA 95064 USA; Department of Ecology and Evolutionary Biology, University of California at Santa Cruz, Santa Cruz, CA 95064 USA; Department of Ecology and Evolutionary Biology, University of California at Santa Cruz, Santa Cruz, CA 95064 USA; Department of Ecology and Evolutionary Biology, University of California at Santa Cruz, Santa Cruz, CA 95064 USA

## Abstract

Garter snakes are semi-aquatic obligate carnivores with diverse diets and behavioral adaptations for foraging at the water’s edge. In this study, we tested the persistence of innate chemosensory prey preference and aquatic prey capture ability in two garter snake species, the Lake Chapala garter snake (*Thamnophis eques obscurus*) and checkered garter snake (*T. marcianus*) that were captive bred and naïve to aquatic prey and aquatic prey capture contexts. Using number of tongue flicks, number of attacks, and attack latency as proxy for interest in terrestrial and aquatic prey extracts, we corroborated dietary preferences reported in the literature that described *T. e. obscurus* as an obligate forager and *T. marcianus* as a facultative aquatic forager in their native habitat. We then categorized strike mode and quantified several metrics describing prey capture efficiency at two treatment depths—shallow (2 cm; wading depth) and deep (10 cm; submerged depth). At shallow depths, *T. e. obscurus* used mostly forward strikes, had a greater gape angle, was more accurate at striking live mosquito fish, was faster at capturing fish prey, and had better visual acuity as indicated by pupil constriction. In shallow water, *T marcianus* used lateral strikes to capture prey. Foraging at increased depth resulted in forward strikes, larger gape angles, longer prey capture latency, longer foraging times, and greater pupil constriction in both species. Moreover, in deeper water, both snake species tended to stay underwater to transport (swallow) prey rather than resurface. We also compared head shape to help explain strike mode and found that *T. e. obscurus* had narrower and shorter heads compared to *T. marcianus* who had wider and taller heads. Our work provides insight into the innate prey preference of naïve snakes and their adaptability to aquatic prey capture contexts. Specifically, we show how an aquatic specialist is more efficient at capturing aquatic prey but that a terrestrial-aquatic generalist is flexible and exhibits behavioral variation across aquatic contexts. Finally, we show how the deep-water context requires behavioral convergence in strike mode, pupil constriction to respond to refractive changes in the water, and tests the ability of semi-aquatic snakes to stay underwater for longer periods of time.

## Introduction

Across secondarily aquatic vertebrates, a wide variety of foraging modes have evolved for targeting aquatic prey, including suspension feeding filter-, particulate-, and bulk-feeding (Crowder 1985; Sanderson and Wassersug 1990), suction feeding (Kienle et al. 2018), engulfing (Naher and Sarker 2014), and jaw prehension (Schwenk and Rubega 2005). Secondarily aquatic vertebrate lineages such as sea snakes (Voris 1983) and semi-aquatic snakes (Young 1991), maintain terrestrial jaw prehension for prey capture underwater (Segall et al. 2019) but have still evolved a myriad of ways for apprehending aquatic prey. Aquatic prey, such as fish, have the ability to sense the water flow of approaching predators through their lateral line system (Stewart et al. 2014), increasing their ability to evade being consumed. Piscivorous semi-aquatic snakes attempt to capture aquatic prey by a bold aerial attack where they watch from above the water’s surface using a craning position (Drummond 1983) so when aquatic prey rapidly approach, their head quickly breaks the surface of the water (Alfaro 2002; personal observations). The tentacled snake (*Erpeton tentaculatum*), a fully aquatic freshwater species conserves energy by omitting the chase and instead, strikes where they predict the fish will move (Catania 2009). Similarly, some semi-aquatic snakes categorized as aquatic specialists use a sit-and-wait approach and use their tongue as a lure (i.e., lingual luring) to attract a prey item toward the predator so the predator can quickly strike (Welsh and Lind 2000).

Semi-aquatic snakes (family Natricidae), such as water snakes (genus *Nerodia*) and garter snakes (genus *Thamnophis*), have been described using: *fast forward* or *frontal strikes* (Alfaro 2002) and *sideways sweeps* or *lateral strikes* (Drummond 1983; Young 1991) when in the water. Common water snakes (*Nerodia sipedon*), use open mouth searching when foraging for fish underwater, a behavior thought to overcome drag (Young 1991). Neonate Diamondback water snakes (*N. rhombifer*) forage primarily on the surface of the water while adults use open-mouthed foraging underwater (Savitzy and Burghardt 2000).

Drummond (1983) showed that aquatic specialists, such as *T. couchii* and *T. melanogaster*, tend to strike from farther away and use forward striking compared to terrestrial-aquatic generalists, such as *T. sirtalis* and *T. elegans*, that use lateral striking and tend to strike from a closer distance. *Thamnophis rufipunctatus*, an aquatic specialist, preferentially performed fast forward strikes when prey was in closer range while *T. sirtalis* performed lateral strikes at prey that were further away (Alfaro 2002). Moreover, as water depth increased, adult *T. validus*, a semi-aquatic generalist, preferentially used fast forward striking (de Queiroz 2003), presumably due to generalist behaviors being less effective at depth compared to specialized ones (Drummond 1983). Both forward- and lateral striking are largely associated with aquatic specialist and terrestrial-aquatic generalist tendencies forming more extremes on a continuum of aquatic behaviors. In addition to strike behavior, snake species that rely more on foraging underwater tend to exhibit a reduction in pupil aperture upon submergence to compensate for the change in refractory index transitioning from air to water to improve focus in the water (Fotenot 2008). This continuum for semi-aquatic snakes warrants more investigation to better capture the behavioral variation of semi-aquatic snakes that regularly move between different water depths and land.

Deep water foraging is a context that has been repeatedly shown to necessitate specialization in snake behavior and morphology. In deeper pools of water, prey have more area to disperse; as predators, snakes must invest more time searching extended areas to locate prey. Aquatic snakes have been shown to converge on a hydrodynamic head shape that is better suited for a forward or frontal strike (Segall et al. 2019). Similarly, aquatic foraging necessitates faster strike speeds (Van Wassenbergh et al. 2009) that overcome the effects of hydrostatic drag associated with greater gape angles required for casting a “wider net” to increase the likelihood of prey capture success (Alfaro 2002). Moreover, foraging at depth requires greater visual acuity for striking agile prey in a greater three-dimensional space (Schaeffel and de Queiroz 1990). Better vision, specifically being able to correct for the refractory index of water permits aquatic specialists, *T. melanogaster* and *T. couchii* to detect and strike prey from greater distances to secure prey capture with precision (Schaeffel and de Queiroz 1990). Given that piscivorous snakes tend to be obligate aquatic foragers and more generalist species facultatively forage in water, the ability to constrict the pupils and have greater refractive power is an anatomical specialization for foraging at depth.

We examined how water depth influences behaviors utilized in aquatic prey capture in two garter snake species with different degrees of aquatic dependency and dietary preference. For this comparison, we selected the Lake Chapala garter snake, *T. eques obscurus*, which is endemic to El Lago de Chapala, Mexico, and the checkered garter snake, *T. marcianus*, found along the Southwestern United States, Northern Mexico, with isolated populations in Central America (Rossman et al. 1996). In the literature, *T. e. obscurus* has only been documented to consume fish found on Lake Chapala, the largest freshwater lake in Mexico, including *Goodea atripinnis, Chapalichthys encaustus, Alloophorus robustus*, and *Poeciliidae infans* (Conant 2003). Consequently, *T. e. obscurus* is an obligate aquatic foraging specialist, constrained to feeding on fish. *Thamnophis marcianus*, on the other hand, is broadly distributed across arid grasslands to the desert, and has been documented to consume a wide variety of terrestrial to aquatic prey including: leeches, worms, lizards, fish, tadpoles, and frogs (Seigel et al. 2000; Heptinstall et al. 2024). Thus, *T. marcianus* is considered a facultative aquatic forager and terrestrial-aquatic generalist (Rayburn 1990)

Chemosensory behavior, or using taste and smell to forage for prey is critical to the foraging ecology of snakes (Arnold 1981; Schwenk 1995; Saviola et al. 2012; Teshera and Clark 2021). Chemosensory studies have been used to determine prey preference of snake species at different life history stages (Burghardt 1970; Arnold 1978; Halpern 1979). Here, we perform two iterative experiments to understand how dietary preference in naïve individuals informs prey capture flexibility in aquatic foraging at different depths. We study naïve, captive-bred juveniles to first determine whether chemosensory preferences coincide with diet records reported for wild individuals of the same species. Second, we assess the aquatic prey capture abilities of those same individuals as naïve adults at two different water depths, shallow and deep. The shallow treatment represented a wading context where snakes are at the water’s edge and the deep represented a context where the snakes would be fully submerged in open water. Testing snakes naïve to chemical prey cues and aquatic foraging contexts will allow us to determine the strength between innate feeding preferences and prey capture strategies in garter snakes and determine whether naïve snakes can exhibit behavioral flexibility in feeding and in which of the two contexts behaviors are more specialized.

## Materials and methods

### Study animals

We used multi-generation captive-bred checkered garter snakes (*Thamnophis marcianus*; 2 males: 5 females) and Lake Chapala garter snakes (*T. e. obscurus*; 3 males: 2 females) raised in the lab since they were 16–20 weeks old under the same environmental conditions and diet. Snakes were housed in plastic enclosures (Sterilite 15 qt, 16 1/4″L x 11 1/4″ × W × 63/4 H) with paper towel substrate, a water dish for drinking, a PVC tube shelter, and aquarium-grade plastic plants for enrichment and climbing. Plastic enclosures were cleaned once a week or more frequently if indicated. Half of each enclosure was placed over a heating pad to establish a temperature gradient from 22 to 30°C. Enclosures were maintained in the laboratory where they were on a 12h:12h light: dark cycle. Prior to experiments, snakes were maintained on a weekly diet of frozen-thawed pinky mice (*Mus musculus*). All experiments and husbandry were conducted at the University of California at Santa Cruz, Coastal Biology Campus and in accordance with the Institutional Animal Care and Use Committee, UCSC protocol number: Mehtr2301dn_r1. Following chemosensory studies, snakes began an alternating diet of pinky mice and Atlantic silverside fish (*Menidia menidia*) that comprised 10% of snake’s body mass, weekly. Snakes are considered naïve in the experimental aquatic foraging context since they were maintained on frozen-thawed prey offered off of tongs in a terrestrial setting until the start of the experiment.

### Prey-preference experimental design

We used tongue-flicking (TF) as a behavioral assay to assess discrimination of prey-related chemical cues. This method uses number of tongue flicks to different chemical stimuli as an indicator of prey preference (Cooper and Burghardt 1990; Cooper and van Wyk 1994; Clark 2004). We selected the following familiar and novel terrestrial and aquatic prey items to extract chemical cues: house mouse (*Mu. musculus*), Atlantic silverside fish (*Me. menidia*), and Canadian nightcrawler worms (*Lumbricus terrestris*). Prey were based on the diets of wild populations reported in the literature and captive snakes were naïve to all prey except for mouse.

Before experimental trials began, snakes were exclusively feeding on mice so fish and worm represented novel prey to naïve snakes at 48 weeks old. We followed Burghardt (1975) for extract preparation and presentation, where 1.5 g of each prey item (mice, fish, or worm) was combined with 10 mL of distilled water. This solution was then heated to 52°C and stirred for 1 min. The prey item was then removed from the solution and the resulting liquid was centrifuged for 15 min at 2000 rpm. Prey remains were strained and the leftover liquid served as the prey extract. Extracts were stored in a freezer and thawed on days when trials were performed. Prey extract order was randomized each week to prevent habituation or bias toward any particular prey item.

Prey extracts, including a distilled H_2_O control, were presented to each snake in an experimental arena for 1 min on a 15-cm long cotton swab. The swab followed the snake as it moved, maintaining a 2-cm distance from the tip of the snout and never touched the snake. Each trial concluded when the 1-min timer ran out or an attack was made. We defined “attack” as opening the mouth and physically striking the prey item in the form of a bite. All 1-min trials were recorded at 60 fps with a GoPro HD Hero Five Camera that provided a dorsal view of the experimental arena. Trials occurred over three weeks with each week consisting of 4 experimental days followed by 3 rest.

In total, 634 trials across the two species over 12 days (*T. e. obscurus*, H_2_O: *N* = 63, worm: *N* = 63, mouse: *N* = 63, fish: *N* = 63; *T. marcianus*, H_2_O: *N* = 84; worm: *N* = 84, mouse: *N* = 84, fish: 84) During video analyses, we recorded the number of tongue flicks oriented toward the cotton swab for each extract and the control, the number of attacks, and attack latency (time to attack).

### Aquatic prey capture experimental design

Snakes were fasted for 7 days prior to trials to ensure individuals were motivated to feed. We examined aquatic prey capture behavior at two depth treatments: shallow (2 cm) and deep (10 cm). The shallow treatment reflects a context where snakes are wading at the water’s edge and the deep treatment represents a context where snakes are submerged in open water. We chose these depths for comparisons with other semi-aquatic species whose aquatic foraging behaviors have been studied. For prey capture trials, each snake was placed into an aquatic experimental arena (20-gallon, tall, glass aquarium) filled to treatment depth. We attached a plastic platform to the tank on the water surface to allow a resting area for snakes. We introduced 10 mosquito fish (*Gambusia affinis*) of similar size (x̄ ± SD = 3.1 ± 2.91, range = 2.6–3.4 mm) in the tank for each trial as previous studies noted that garter snakes detect prey best when prey aggregate (Alfaro 2002). The prey density was maintained across treatments.

For shallow-water treatments, trials commenced when individual snakes were placed in the center of the tank whereas in deep-water treatments, individual snakes were placed on the terrestrial platform. Trials ended for both treatments when individuals had a successful prey capture event or a snake either musked or tried to escape the experimental arena both of the latter behaviors indicating that the individual was not motivated to feed. Only trials where a successful prey capture event occurred were analyzed.

All aquatic prey-capture trials were filmed with a dorsal and lateral view with two cameras (models: GoPro HD, Hero 5 and 9) at 60 fps. We analyzed video for the following prey capture behaviors: gape angle, strike mode, number of strikes prior to a successful prey capture, prey capture latency, prey body part captured (head, midsection of the body or “body” herein, or tail), prey transport (swallowing) location (underwater, while resurfacing), prey transport time, and total time underwater.

We adopted aquatic strike mode terminology following (Drummond 1983; Alfaro 2002): *forward strikes*, where the snake strikes head-on at the oncoming prey within the water, and *lateral strikes*, also referred to as sideways sweeps, where the snake strikes from a lateral angle. For measuring gape angle, video stills were captured during successful trials where the snake was in a lateral view and then imported into a digital protractor platform (protactoronline.com) for measurement. Each image measured was recorded as one data point.

### Statistical analyses

Statistical analyses were conducted using R version 4.5.0 (2025–04–11). We tested for normality in our continuous data to determine whether interest in prey extracts (number of tongue flicks, number of attacks, and attack latency) and prey capture data (gape angle, number of strikes prior to success, prey capture latency, prey transport time, and pupil diameter) followed a normal distribution. Number of tongue flicks, attack latency, and total attacks failed normality according to our Shapiro–Wilk tests. Therefore, we used Kruskall–Wallis tests to determine whether snakes discriminated between prey chemical extracts.

For aquatic prey-capture data, gape angle followed a normal distribution enabling us to use a repeated measures analysis of variance (ANOVA) to test for differences between shallow and deep-water treatments. Number of strikes, prey capture latency, prey transport time, and total time underwater were not normally distributed. Consequently, Kruskal–Wallis tests were performed for statistical comparisons to examine the relationship between non-normally distributed prey capture metrics for both species at the two depth treatments. Secondarily, Wilcoxon signed-rank tests were conducted for non-normal data and low sample sizes. When determining statistical significance for proportions of strike mode, prey capture position, and prey capture location against species and depth treatment, we used a Pearson’s Chi-squared with Yate’s continuity correction test. Significant differences in prey-preference experiments and aquatic prey capture treatments were followed with Tukey’s post-hoc tests to determine significance between pair-wise comparisons.

We measured pupil diameter as a proxy for visual acuity or how clear snakes could see underwater. We measured relative difference in pupil diameter when snakes were in air and while foraging at treatment depth. We took video stills during successful trials where the snake was in a lateral view in air and underwater in the same plane (against the glass closest to the viewer). These images were then imported into the open-source image analyzer program, ImageJ. Due to the nature of eye and pupil diameter distance varying between images, rather than measure eye diameter directly, we measured total eye diameter and pupil diameter to obtain a proportion for each snake in air and in water at the two depths, shallow and deep. We then compared the relative proportions of the pupil in air, shallow, and deep water.

For presentation of results, we also ran linear mixed effects models where species (*T. e. obscurus* and *T. marcianus*) and depth treatments (shallow and deep) were fixed effects and individual ID was used as a random effect. We also report Cohen’s effect sizes to showcase degree of significance (small, medium, and large).

For head shape, we took dorsal, lateral, and ventral photographs and analyzed morphological standard lengths and ratios according to Deepak et al. (2023). Dorsal measurements included head width between anterodorsal corners of first supralabials (HWFSL), length between nares (NN), length between eyes (EE), head width (HW), and head width between posterodorsal corners of last supralabials (HWLSL). Lateral measurements included length between eye and snout (ES), length between eye and nare (EN), eye diameter (ED), head height (HH), head height between parietals (HHP), length between nare and lip (NL), length between center of eye and lip (EL), head length from snout to retroarticular process (HLRA), and ventral (JL), and head length from snout to first ventral (HLV). Following Deepak et al. (2023), we also used log-shape ratios (Mosimann 1970) and divided the 15 head measurements to obtain comparable ratios in the head shape descriptors of interest (higher-, lower-, wide-, and narrow heads; short vs. long eye to nare lengths).

## Results

### Prey preference in juvenile snakes

In total, we recorded 634 trials over 12 days for both species but 576 of the trials yielded results (240 trials for *T. e. obscurus*, H_2_O: *N* = 60, worm: *N* = 60, mouse: *N* = 60, fish: *N* = 60; 336 trials for *T. marcianus*, H_2_O: *N* = 84; worm: *N* = 84, mouse: *N* = 84, fish: 84).

#### Number of tongue flicks

**Fig. 1:**
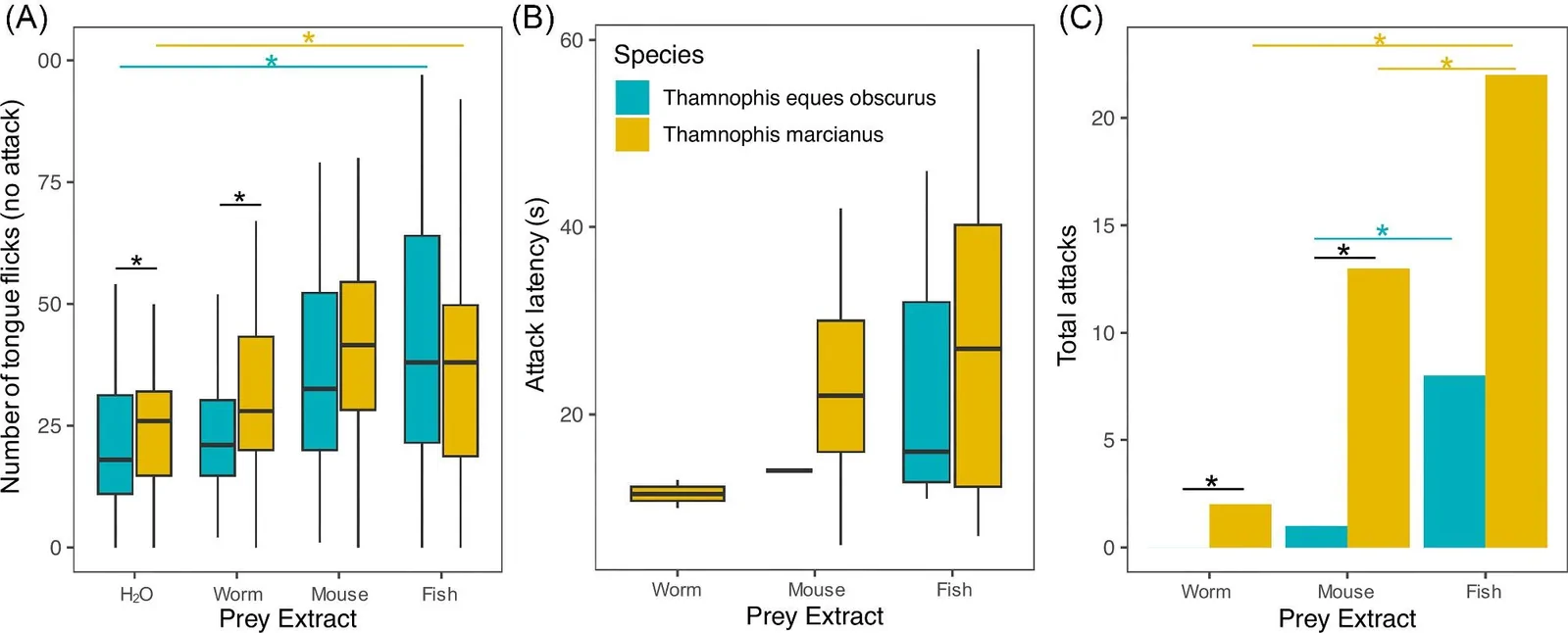
Prey preference metrics for naïve neonate *T. e. obscurus* and *T. marcianus* exposed to different prey extracts. The black central line represents the median. (A) Number of tongue flicks. (B) Attack latency. Distilled water (H_2_O) was omitted since it was not attacked. (C) Total attacks. Distilled water (H_2_O) was omitted since it was not attacked. Asterisks denote significant differences (*P* < 0.05).

The number of tongue flicks (TF) elicited by prey extracts and the control for both species varied significantly across treatments (Table 1: *T.e.o*: *P* < 0.001; *T.m.*: *P* < 0.001). Fish averaged the highest number of tongue flicks for *T. e. obscurus* (Table 2: x̄ = 40.58), while mouse averaged the highest number of tongue flicks for *T. marcianus* (x̄ = 39.77). *Thamnophis marcianus* exhibited heightened tongue flicks for distilled water (*P* = 0.047) and worm (*P* = 0.006) relative to *T. e. obscurus* (Fig. 1A). When comparing species tongue flick responses toward the three extracts and distilled water, there were no significant differences between the two species. Pairwise comparisons did not detect differences between species for mouse (*P* = 0.25) or fish (*P* = 0.27). A linear mixed effects model where individual is a random factor and species and prey extract revealed that tongue flicks significantly increased for mouse (∼15 TF; *P <* 0.001) and fish (∼14 TF; *P* < 0.001) and moderately increased for worm (∼4 TF; *P* = 0.04). Species-level differences were not significant under this model (*P* = 0.529). Cohen’s *d* effect sizes yielded that species was negligible (*d* = −0.1061447; 95% CI: lower: −0.27225553, upper: 0.05996609).

**Table 1:** Summary table for prey preference comparisons for *T. e. obscurus* and *T. marcianus*.

Predictors	Number of tongue flicks (no attack)	Attack latency (s)	Total attacks
	*P*	*P*	*P*
Tests	Kruskal–Wallis	Kruskal–Wallis (Wilcoxon)	Kruskal–Wallis
**H_2_O:Species**	**0.047** *	**N/A**	**N/A**
**H_2_O:Worm:Species**	0.92	**N/A**	**N/A**
*Thamnophis eques obscurus*	**0.39**	**N/A**	**N/A**
*Thamnophis marcianus*	**0.41**	**N/A**	**N/A**
**H_2_O:Mouse:Species**	0.5	N/A	N/A
*Thamnophis eques obscurus*	0.46	N/A	N/A
*Thamnophis marcianus*	0.85	N/A	N/A
**H_2_O:Fish:Species**	0.53	N/A	N/A
*Thamnophis eques obscurus*	0.31	N/A	N/A
*Thamnophis marcianus*	0.59	N/A	N/A
**H_2_O:Worm:Mouse:Fish:Species**	N/A	N/A	N/A
*Thamnophis eques obscurus*	**<0.001** ***	N/A	N/A
*Thamnophis marcianus*	**<0.001** ***	N/A	N/A
**Worm:Species**	**0.006** *	0.46	0.031*
**Worm:Mouse:Species**	0.5	N/A	0.45
*Thamnophis eques obscurus*	0.64	N/A	N/A
*Thamnophis marcianus*	0.63	N/A	0.9
**Worm:Mouse:Fish:Species**	N/A	N/A	N/A
*Thamnophis eques obscurus*	N/A	N/A	N/A
*Thamnophis marcianus*	N/A	0.31	**0.02** *
**Worm:Fish:Species**	0.47	N/A	N/A
*Thamnophis eques obscurus*	0.66	N/A	N/A
*Thamnophis marcianus*	0.35	N/A	0.02*
**Mouse:Species**	0.25	−0.46	0.012*
**Mouse:Fish:Species**	0.29	N/A	**<0.001** ***
*Thamnophis eques obscurus*	0.53	**0.7**	**<0.001** ***
*Thamnophis marcianus*	**0.068**	**N/A**	0.014*
**Fish:Species**	**0.27**	**−0.71**	0.11

*P < 0.05, **P < 0.01, ***P < 0.001.

**Table 2:** Means for prey preference for *T. e. obscurus* and *T. marcianus*.

	*Thamnophis eques obscurus*				*Thamnophis marcianus*			
Prey	H_2_O	Worm	Mouse	Fish	H_2_O	Worm	Mouse	Fish
Preference	$\bar{\rm x}$ ± SD	$\bar{\rm x}$ ± SD	$\bar{\rm x}$ ± SD	$\bar{\rm x}$ ± SD	$\bar{\rm x}$ ± SD	$\bar{\rm x}$ ± SD	$\bar{\rm x}$ ± SD	$\bar{\rm x}$ ± SD
Parameters	Range	Range	Range	Range	Range	Range	Range	Range
Number of tongue flicks	21.22 ± 13.68	24.02 ± 12.75	36.23 ± 20.99	40.58 ± 26.16	25.01 ± 11.20	30.60 ± 15.95	39.77 **±** 22.77	35.19 **±** 22.98
	0–54	2–58	1–79	0–97	0–50	0–67	0–96	0–92
Total attacks	0	0	1	8	0	2	13	22
Attack latency	N/A	N/A	14	23 ± 13.90	N/A	11.5 ± 2.12	23.23 ± 10.18	26.82 ± 14.98
				11–46		10–13	6–42	7–59

#### Attack latency

Snakes did not vary their attack latency across prey extracts (Fig. 1B). Prey items of interest were attacked within 22 s (*T. e. obscurus*.) and 25 s for (*T. marcianus*) for the 1-min trial. Similarly non-significant results were found under a linear mixed effects model where individual was treated as a random factor and species and prey extract were fixed effects. Cohen’s *d* effect sizes were small for species (*d* = −0.2041943; 95% CI: lower: 0.9544725, upper: 0.5460839).

#### Number of attacks

Snake species did not attack prey extracts equally. Neither species attacked the distilled H_2_O control. *Thamnophis e. obscurus* never attacked swabs with worm extract while *T. marcianus* attacked swabs with worm extract twice (*P* = 0.031; Table 2; Fig. 1C). *Thamnophis marcianus* made significantly more attacks on swabs with mouse extract (*N* = 13) than *T. e. obscurus* (*N* = 1; *P* = 0.012). Similarly, there were species-level differences across mouse and fish (*P* < 0.001) where *T. e. obscurus* (*P* < 0.001) attacked more swabs with fish extract (*N* = 8) than mouse extract (*N* = 1) compared to *T. marcianus* (*P* = 0.014) who made more attacks on mouse (*N* = 13) and fish (*N* = 22) overall. Attacks made toward worm and mouse extracts by *T. marcianus* were not significantly different (*P* = 0.9) but pairwise comparisons showed differences between fish and worm extract (*P* = 0.02) with fish extract eliciting the most attacks. With a linear mixed effects model where individual is a random effect, prey extract attacks on fish (*P* < 0.001) and mouse (*P* < 0.01) were significant. There were no species-level significant results found under this model. Cohen’s *d* effect sizes resulted in a small effect size for species (*d* = −0.2792422; 95% CI: lower: −0.4436901, upper: −0.1147944).

### The effects of treatment depth on prey capture behavior in naïve adult snakes

In total, we analyzed 70 trials (18 trials for *T. e. obscurus*, shallow: *N* = 4; deep: *N* = 14; 52 trials for *T. marcianus*, 2 cm shallow: *N* = 22; deep: *N* = 30; see Table 3 for a summary of prey capture behavior means). We quantify the metric “efficiency” as the relative time it took for the two species to successfully capture prey. A more efficient forager would take less time which would presumably be associated with lower energetic costs. Similarly, we quantified accuracy by comparing the number of strikes snakes took to successfully capture prey with less strikes suggesting higher accuracy during foraging.

**Table 3:** Means for prey capture for *T. e. obscurus* and *T. marcianus*.

	*Thamnophis eques obscurus*						*Thamnophis marcianus*					
Prey	Shallow			Deep			Shallow			Deep		
Capture	$\bar{\rm x}$ ± SD			$\bar{\rm x}$ ± SD			$\bar{\rm x}$ ± SD			$\bar{\rm x}$ ± SD		
Parameters	Range			Range			Range			Range		
Strike mode proportion and transport time	**Forward**		**Lateral**	**Forward**		**Lateral**	**Forward**		**Lateral**	**Forward**		**Lateral**
	*N* = 8 (67%)		*N* = 4 (33%)	*N* = 12 (67%)		*N* = 6 (33%)	*N* = 10 (21%)		*N* = 38 (79%)	*N* = 26 (67%)		*N* = 13 (33%)
	11.38 ± 11.61		51 ± 69.91	15.67 ± 11.49		17.33 ± 10.86	18.90 ± 15.24		22.55 ± 41.09	15 ± 11.69		18.62 ± 24.60
	3–37		**9–155**	3–37		5–30	6–55		4–186	5–47		4–93
Maximum gape angle (°)	44 ± 11.05			46.93 ± 10.77			23.05 ± 5.07			25.97 ± 6		
	31–58			30–66			15–34			16–40		
Number of strikes	1.42 ± 1.16			1.5 ± 0.62			2.94 ± 2.47			2.72 ± 2.44		
	1–5			1–3			1–11			1–11		
Prey capture latency (s)	3.82 ± 5.56			5.53 ± 3.34			11.22 ± 13.63			27.31 ± 28.36		
	1–16			1–13			1–77			2–121		
Prey transport time (s)	24.58 ± 42.42			16.22 ± 10.99			21.79 ± 37.09			16.21 ± 16.85		
	3–155			3–37			4–186			4–93		
Total time underwater (s)	10 ± 6.97			25.53 ± 14.55			21.80 ± 20.79			84.31 ± 69.76		
	2–21			6–51			2–101			3–304		
Prey capture position proportion and transport time (s)	**Head**	**Body**	**Tail**	**Head**	**Body**	**Tail**	**Head**	**Body**	**Tail**	**Head**	**Body**	**Tail**
	*N* = 5 (42%)	*N* = 4 (33%)	*N* = 3 (25%)	*N* = 6 (33%)	*N* = 5 (28%)	*N* = 7 (39%)	*N* = 8 (17%)	*N* = 12 (25%)	*N* = 28 (58%)	*N* = 9 (23%)	*N* = 17 (44%)	*N* = 13 (%)
	5 ± 3.46	57.75 ± 65.81	13 ± 4	5.33 ± 2.58	20.2 ± 11.52	22.71 ± 8.20	15.13 ± 25.8	10.5 ± 3.90	28.54 ± 45.92	15.89 ± 17.52	14.82 ± 20.28	18.23 ± 11.78
	3–11	10–155	9–17	3–10	8–37	12–31	4–77	6–20	4–186	4–47	6–93	7–42
Prey transport proportion and location time (s)	**Resurfacing**		**Underwater**	**Resurfacing**		**Underwater**	**Resurfacing**		**Underwater**	**Resurfacing**		**Underwater**
	*N* = 7 (58%)		*N* = 5 (42%)	*N* = 3 (17%)		*N* = 15 (83%)	*N* = 36 (75%)		*N* = 12 (25%)	*N* = 5 (13%)		*N* = 34 (87%)
	38.29 ± 52.54		5.40 ± 4.34	23 ± 15.72		14.87 ± 9.98	26 ± 42.05		9.17 ± 4.61	33 ± 36.38		13.74 ± 10.84
	9–155		3–13	6–37		3–31	4–186		4–16	6–93		4–47
Percent of pupil constriction	54.51 ± 6.22			46.76 ± 4.89			66.53 ± 5.40			61.60 ± 5.49		
	46.15–63.73			40.57–58.67			56.25–80.00			50.38–71.88		

#### Strike mode

**Fig. 2:**
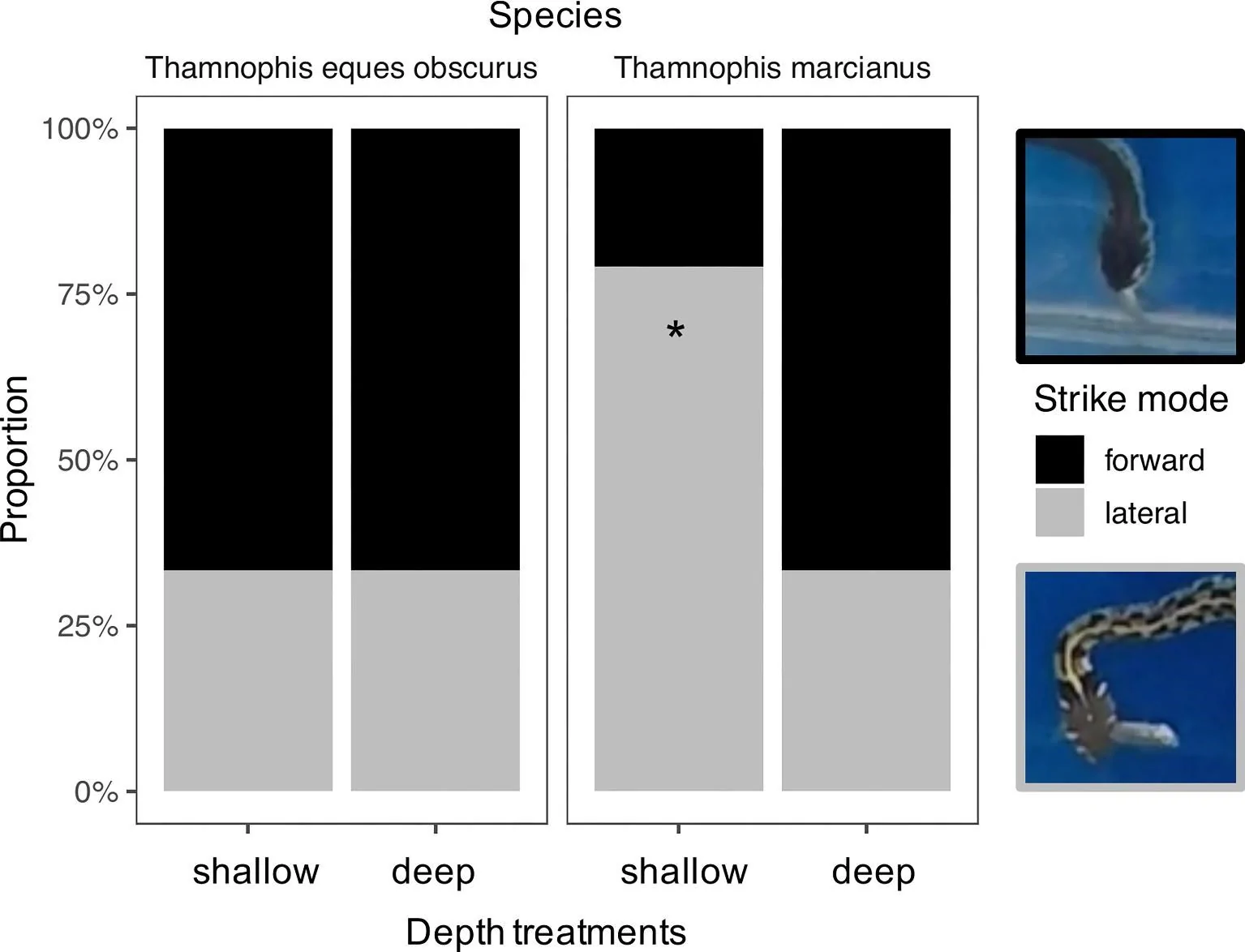
Proportion of strike modes (forward and lateral) at two treatment depths: shallow (wading; 2 cm) and deep (submerged; 10 cm) for *T. e. obscurus* and *T. marcianus*. While forward striking was more common for *T. e. obscurus* at both depths, *T. marcianus* showed more flexibility across depth and converged on forward striking in deeper water. Asterisks denote significant differences (*P* < 0.05).

Species (Table 4: *P* = 0.029) and treatment depth (*P <* 0.001) were significant predictors of strike mode. *Thamnophis marcianus* used lateral strikes in shallow water but preferentially used forward strikes in deep water (*P <* 0.001). *Thamnophis e. obscurus* used forward strikes in both shallow and deep water with no significant difference between treatment depth (*P* > 0.05). Both species exhibited similar proportions of forward striking in deep water (Fig. 2: *T. m*.: 67.7%, *N* = 26; *T. e. o*.: 67.7%, *N* = 12).

**Table 4:** Pearson’s Chi-squared with Yate’s continuity correction test summary table for proportion of strike mode, prey capture position, and prey capture location at both depth treatments for *T. e. obscurus* and *T. marcianus*.

*Predictors*	*X^2^*	*df*	*P*
**Strike mode:Species**	**4.75**	**1**	0.029*
**Strike mode:Depth:Speices**	**14.31**	**1**	<0.001***
*Thamnophis eques obscurus*	0	1	1
*Thamnophis marcianus*	**16.79**	**1**	<0.001***
**Strike mode:Shallow:Species**	**7.55**	**1**	0.006**
**Strike mode:Deep:Species**	**0**	**1**	**1**
**Prey capture position:Species**	**3.78**	**2**	0.15
**Prey capture position:Depth:Species**	**3.39**	**2**	0.18
*Thamnophis eques obscurus*	0.63	2	0.73
*Thamnophis marcianus*	**5.54**	**2**	0.06
**Prey capture position:Shallow:Species**	**5.084**	**2**	0.079
**Prey capture position:Deep:Species**	**1.4**	**2**	0.5
**Prey capture location:Species**	**1.21**	**1**	**0.27**
**Prey capture location:Depth:Species**	**37.18**	**1**	<0.001***
*Thamnophis eques obscurus*	**3.91**	**1**	0.048*
*Thamnophis marcianus*	**30.94**	**1**	<0.001***
**Prey capture location:Shallow:Species**	**0.62**	**1**	**0.43**
**Prey capture location:Deep:Species**	**<0.001**	**1**	**1**

*P < 0.05,  **P < 0.01,  ***P < 0.001.

#### Gape angle

**Fig. 3:**
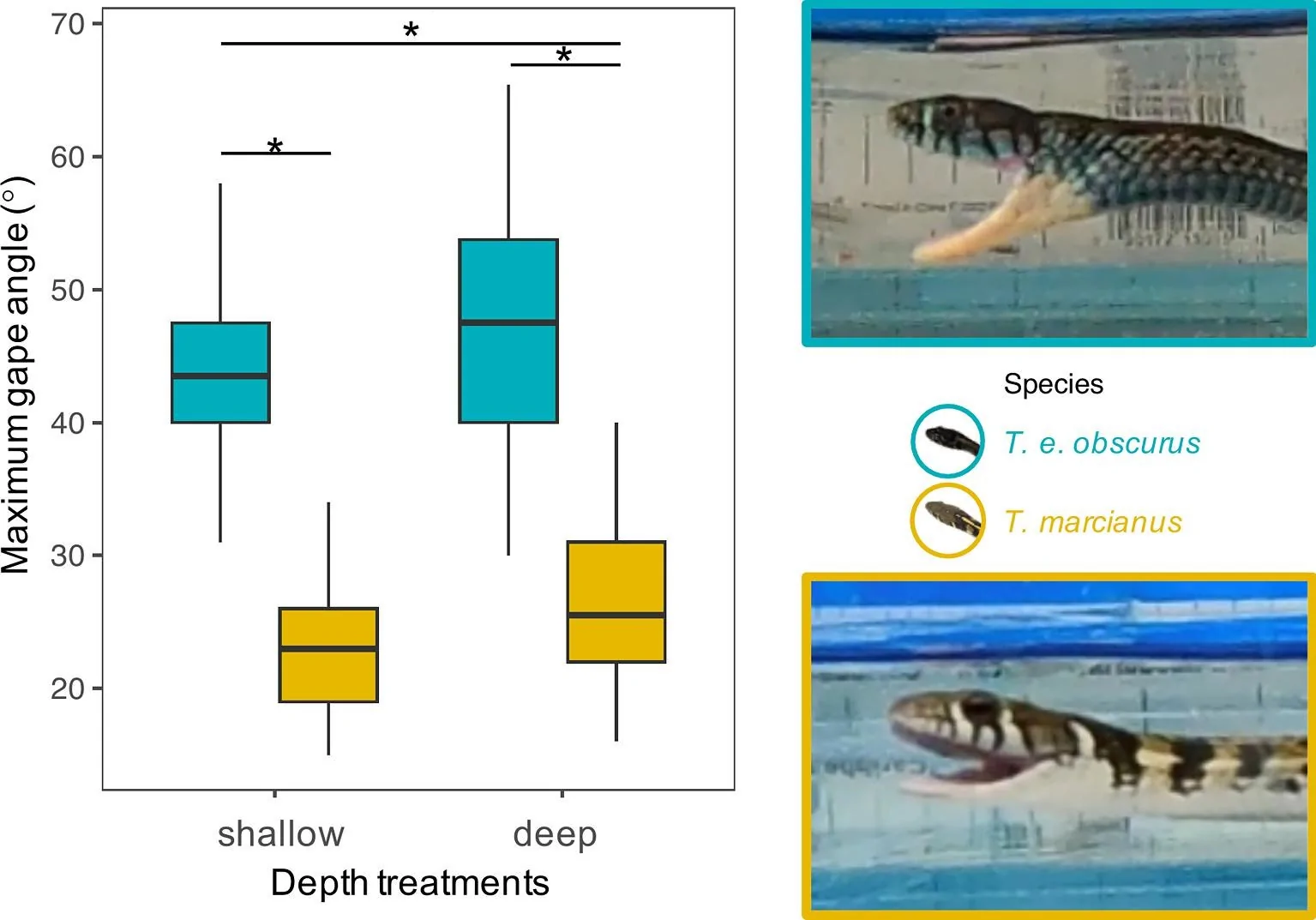
Gape angle during prey capture for *T. e. obscurus* and *T. marcianus*. (A) Maximum gape angle (degrees) directly leading to a successful prey capture at two depth treatments: shallow (wading; 2 cm) and deep (submerged; 10 cm). (B) Video still of maximum gape for *T. e. obscurus* at 2 cm. (B) Video still of maximum gape for *T. marcianus* at 2 cm. Asterisks denote significant differences (*P* < 0.05).

We analyzed gape angle for 72 prey capture events across the two species (*N* = 20; *T. e. obscurus*, shallow: *N* = 9; deep: *N* = 11; *T. marcianus*, 2 cm shallow: *N* = 29; deep: *N* = 23). Both treatment depth (*P* = 0.001) and species (*P* < 0.001) were significant predictors for maximum gape angle (Table 5). Specifically, maximum gape angles were larger at the 10 cm depth treatment compared to the shallow depth treatment. Between species, *T. e. obscurus* consistently showed greater maximum gape angles prior to prey capture in both treatment depths (Fig. 3; shallow: x̄ = 44.00; deep: x̄ = 46.93; *T. marcianus*; shallow: x̄ = 23.05; deep: x̄ = 25.97). The greatest gape angle recorded was 66° for *T. e. obscurus* at the 10 cm treatment depth and the smallest gape angle recorded was 15° for *T. marcianus* at the 2 cm treatment depth. No significant differences for maximum gape angle were detected between forward or lateral strike modes. When considering individual ID as a random effect in a linear mixed model, species is a significant predictor (*P <* 0.01) with an average of 23.5° difference in gape angle between *T. e. obscurus* and *T. marcianus* while depth is not (*P* = 0.117), having roughly a 2.67° difference between shallow and deep water treatments. In terms of Cohen’s *d* effect sizes, species had a large effect (*d* = 2.899961; 95% CI: lower: 2.177675, upper: 3.622248) whereas depth had a medium effect (*d* = 0.5118451; 95% CI: lower: 0.01667539, upper: 1.00701481).

**Table 5:** ANOVA summary table for determining the effects of species, depth, and their interactions on maximum gape angle and percent of eye diameter in air for *T. e. obscurus* and *T. marcianus*.

	Maximum gape angle (°)	Percent of eye diameter in air (%)
*Predictors*	*P*	*P*
**Depth**	0.001**	<0.001***
**Depth:Species**	0.778	0.33
*Thamnophis eques obscurus*	**0.64**	0.0058**
*Thamnophis marcianus*	**0.07**	0.0021**
Residuals		
**Species**	<0.001***	<0.001***
Observations	71	72
*R* ^2^ /*R*^2^ adjusted	0.646/0.631	0.632/0.616

*P < 0.05,  **P < 0.01,  ***P < 0.001.

#### Number of strikes

**Fig. 4:**
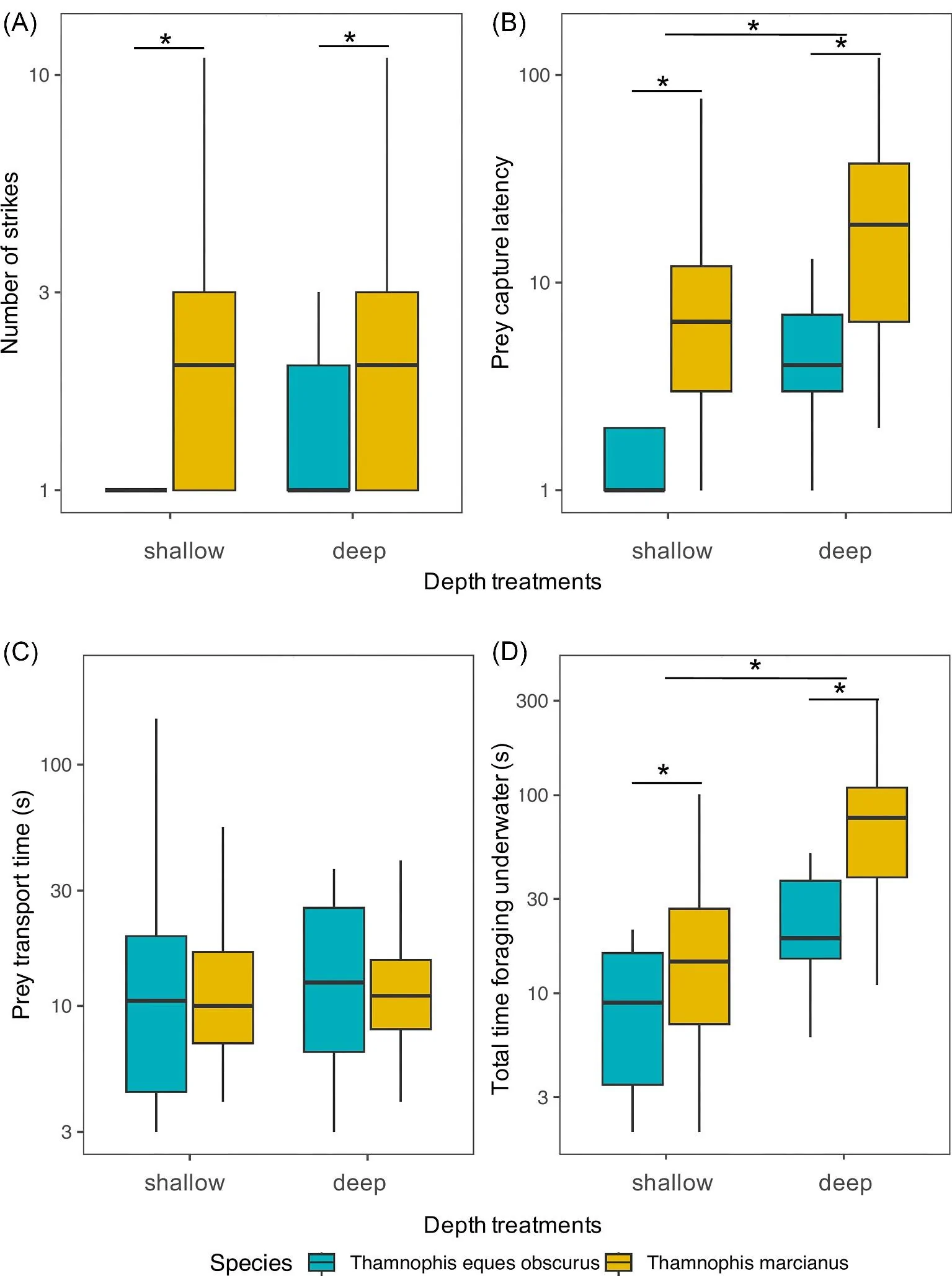
Prey capture metrics at two depth treatments: shallow (wading; 2 cm) and deep (submerged; 10 cm) in *T. e. obscurus* and *T. marcianus*. (A) Number of strikes before prey capture. (B) Prey capture latency. (C) Prey transport time upon prey capture. (D) Total time underwater during foraging bout. Asterisks denote significant differences (*P* < 0.05).

Species was a significant predictor for number of strikes in both shallow (*P* = 0.003) and deep (*P* = 0.046) treatments. *Thamnophis e. obscurus* used fewer strikes to capture prey than *T. marcianus* across treatments (Fig. 4A; Table 6). In a linear mixed effects model with individual as a random factor, species was significant (*P* < 0.05), while depth was not (*P* = 0.7299). Under this model, *T. e. obscurus* took on average 1 strike less to capture prey successfully than *T. marcianus*.

**Table 6:** Summary table for prey capture comparisons for *T. e. obscurus* and *T. marcianus*.

*Predictors*	Number of strikes *P*	Prey capture latency (s) *P*	Prey transport time (s) *P*	Total time underwater (s) *P*
Tests	Kruskal–Wallis (Wilcoxon)	Kruskal–Wallis (Wilcoxon)	Kruskal–Wallis (Wilcoxon)	Kruskal–Wallis (Wilcoxon)
**Depth:Species**	(0.41)	(0.002**)	**(0.52)**	(<0.001***)
*Thamnophis eques obscurus*	**(0.18)**	(0.012*)	**(0.61)**	(0.006**)
*Thamnophis marcianus*	**(0.41)**	<0.001***	**(0.68)**	(<0.001***)
**Shallow:Species**	0.003**	0.002**	**0.84**	0.037*
**Deep:Species**	0.046*	<0.001***	**0.7**	<0.001***

*P < 0.05, **P < 0.01, ***P < 0.001.

#### Prey capture latency

Treatment depth (*P* = 0.002) and species (shallow: *P* = 0.002; deep: *P* < 0.001) were significant predictors for prey capture latency (Table 6). At greater depth, both *T. e. obscurus* (*P* = 0.012) and *T. marcianus* (*P* < 0.001) took longer to capture prey (Fig. 4B). *Thamnophis e. obscurus* was faster and therefore more efficient at capturing prey than *T. marcianus* in both treatments. Running a linear mixed effects model where individual is a random effect and both species and depth are fixed effects yielded significant results for species (*P* < 0.001) and depth (*P* < 0.001). On average, *T. marcianus* captured prey 16 s after *T. e. obscurus* and in terms of depth, deeper water took an additional 13 s to capture prey compared to shallow water. Regarding Cohen’s *d* effect sizes, both species (*d* = –0.6833075; 95% CI: lower: −1.1243784, upper: −0.2422365) and depth had a medium effect (*d* = 0.5386128; 95% CI: lower: 0.1590787, upper: 0.9181470).

#### Prey transport time and the effects of prey capture position and location

**Fig. 5:**
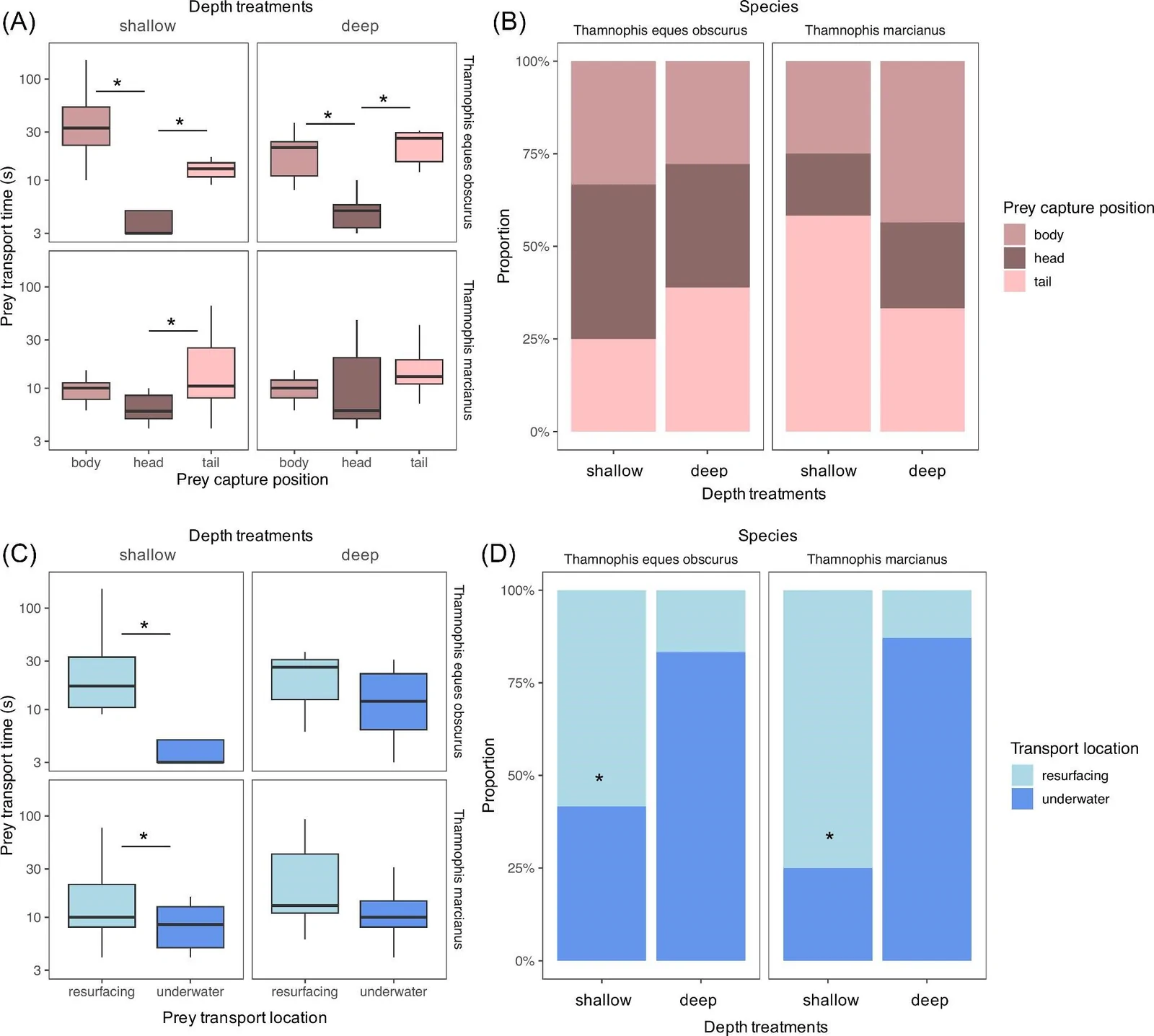
Prey transport time at two depth treatments: shallow (wading; 2 cm) and deep (submerged; 10 cm) for *T. e. obscurus* and *T. marcianus.* (A) Prey transport time (time to swallow prey) relative to prey capture position (region along body where prey was captured). (B) Proportion of prey captured at three different positions. (C) Prey transport time relative to prey transport location. (D) Proportion of prey transported (swallowed) while resurfacing or underwater. Asterisks denote significant differences (*P* < 0.05).

There were no significant predictors for transport time (time to swallow prey) between treatment depth or species (Fig. 4C, Table 6). However, there were significant species-level differences amongst transport times relative to prey capture position (head, body, and tail) and location during transport (resurfacing: underwater) within both treatment depths (Fig. 4A; Table 7). No significant differences were found between the proportion of trials snakes captured prey by the head, body, or tail at either treatment, shallow or deep (Fig. 5B).

**Table 7:** Summary table for determining the effects of prey capture position, prey capture location, and their interactions on prey transport time at both depth treatments for *T. e. obscurus* and *T. marcianus*.

Depth treatment	Shallow	Deep
	**Prey transport time (s)**	**Prey transport time (s)**
*Predictors*	*P*	*P*
**Tests**	**Kruskal–Wallis**	**Kruskal–Wallis**
	**(Wilcoxon)**	**(Wilcoxon)**
**Body:Head:Tail:Species**	**0.027**	**0.051**
*Thamnophis eques obscurus*	0.027*	0.004**
*Thamnophis marcianus*	0.051	**0.073**
**Body:Species**	0.028*	**0.11**
**Body:Head:Species**	(0.003**)	(0.006**)
*Thamnophis eques obscurus*	0.034*	0.013*
*Thamnophis marcianus*	**0.063**	**0.19**
**Body:Tail:Species**	**−0.81**	(0.012*)
*Thamnophis eques obscurus*	**0.23**	**0.53**
*Thamnophis marcianus*	**0.3**	0.032*
**Head:Species**	**0.12**	**0.21**
**Head:Tail:Species**	(0.001**)	(0.002**)
*Thamnophis eques obscurus*	**0.067**	0.003**
*Thamnophis marcianus*	0.029*	**0.17**
**Tail:Species**	**0.92**	**0.2**
**Resurfacing:Underwater:Species**	(0.003**)	−0.15
**Resurfacing:Species**	0.15	0.76
*Thamnophis eques obscurus*	0.018*	0.31
*Thamnophis marcianus*	0.073	0.22
**Underwater:Species**	0.054	0.66

*P < 0.05, **P < 0.01, ***P < 0.001.

In shallow water, species (*P* = 0.027) was a significant predictor for transport time for prey caught by the head, body, or tail for *T. e. obscurus* (*P =* 0.027). Fish prey captured by the body (*P* = 0.028) was transported faster by *T. marcianus* compared to *T. e. obscurus*. There was also a species-level difference for prey transport when comparing prey caught by the body or the head (*P* = 0.003). *Thamnophis e. obscurus* transported prey captured head-first significantly faster than prey caught by the body. Prey transport times for *T. marcianus* were similar between prey caught by the head and body. Finally, at shallow depth, capture position at the head or tail was a significant predictor (*P* = 0.001) of transport time where *T. marcianus* transported prey caught by the head more rapidly than by the tail (*P* = 0.029).

For the 10 cm water treatment, there was a significant difference in transport times between prey captured at the head, body, and tail for *T. e. obscurus* (*P* = 0.004) compared to *T. marcianus*. Species-level differences for prey captured at the head or body (*P* = 0.006) revealed that *T. e. obscurus* transported prey caught by the head significantly faster than prey caught by the body (*P* = 0.013). Similarly, a species-level difference for head and tail (*P* = 0.002) revealed that *T. e. obscurus* transported prey caught by the head more rapidly than prey caught by the tail (*P* = 0.003). Finally, a species-level difference (*P* = 0.012) showed that *T. marcianus* transported prey caught by the body more rapidly than prey caught by the tail (*P* = 0.032).

In the shallow treatment, a significant species-level difference was detected for transporting prey while resurfacing or underwater (*P* = 0.003). *Thamnophis e. obscurus* transported prey more rapidly when remaining underwater compared to when resurfacing (*P* = 0.018). In the deep-water treatment, prey transport times were similar for both species whether snakes were either resurfacing or remaining underwater (Fig. 5C).

Species differed in the proportion of prey transport trials that took place while resurfacing or remaining under water across treatment depth (*P* < 0.001). Both *T. e. obscurus* (58.3%) and *T. marcianus* (75%) preferentially resurfaced to transport prey at the shallow depth (Fig. 5D) while both species, *T. marcianus*: (87.2%) and *T. eques obscurus* (83.3%), preferentially transported prey underwater at depth.

With regards to treating individual ID as a random effect in a linear mixed effects model, both species (*P* = 0.994) and depth (*P* = 0.120), were not significant. Cohen’s *d* effect sizes describe depth as having a small effect (*d* = −0.2109893; 95% CI: lower: −0.5783785, upper: 0.1563998), while species has a negligible effect (*d* = 0.009545022; 95% CI: lower: −0.4098438, upper: 0.4289338).

#### Total time underwater

Treatment (*P* < 0.001) and species (shallow: *P* = 0.037; deep: *P* < 0.001) were significant predictors for time snakes spent underwater. Both snakes spent more time foraging underwater in the deep-water treatment compared to the shallow-water treatment. However, between species, *T. e. obscurus* spent less time underwater in each treatment (Fig. 4D: *P =* 0.006) whereas *T. marcianus* (*P <* 0.001) spent more time foraging underwater. Regarding a linear mixed effects model where individual was treated as a random effect, depth was a significant factor (*P <* 0.001) with deep foraging dives taking approximately 49 s longer than shallow dives, while species was not (*P* = 0.062). Cohen’s *d* effects size show that depth has a large effect (*d* = 0.9882854; 95% CI: lower: 0.5933481, upper: 1.3832227).

#### Pupil constriction underwater

**Fig. 6:**
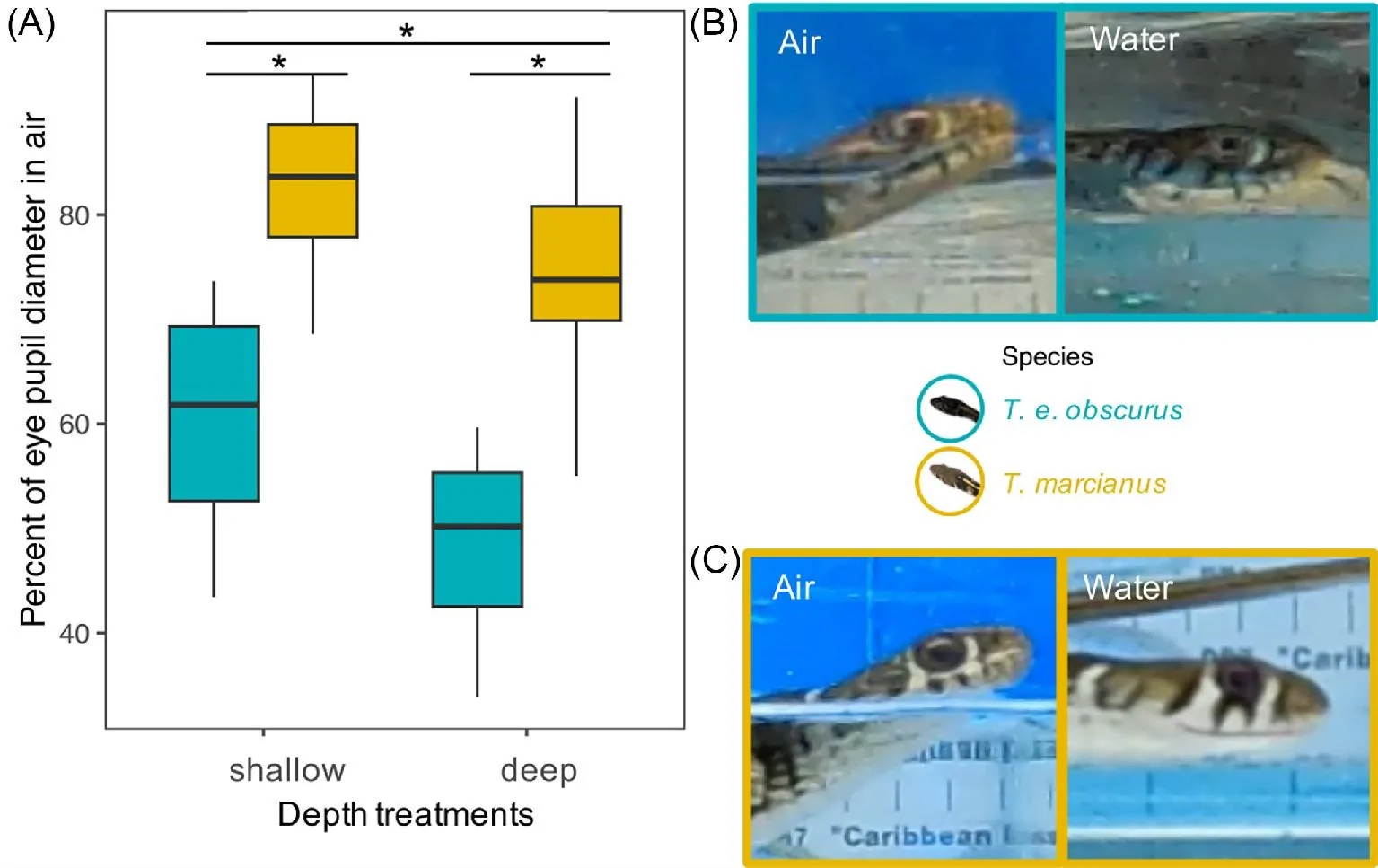
Pupillary diameter in *T. e. obscurus* and *T. marcianus*. (A) Percent of eye pupil diameter in air when underwater during successful prey capture at two depth treatments: shallow (wading; 2 cm) and deep (submerged; 10 cm). (B) Video stills of eye diameter in air and underwater by *T. e. obscurus* at 2 cm. (B) Video stills of eye diameter in air and underwater by *T. marcianus* at 2 cm. Asterisks denote significant differences (*P* < 0.05).

We analyzed relative pupil constriction underwater as a proxy for visual acuity for 72 prey capture events across the two species (*T. e. obscurus*, shallow: *N* = 6; deep: *N* = 14; *T. marcianus*, 2 cm shallow: *N* = 22; deep: *N* = 30). Both treatment depths (*P* = 0.001) and species (*P* < 0.001) were significant predictors for relative pupil constriction (Table 5). Specifically, the proportion of pupil diameter in air was greater at the 10 cm depth treatment compared to the shallow depth treatment. Between species, *T. e. obscurus* consistently showed greater pupil constriction underwater during successful prey capture in both depth treatments (Fig. 6; shallow: x̄ = 59.28; deep: x̄ = 49.08). Individuals of *T. marcianus* (shallow: x̄ = 82.75; deep: x̄ = 74.81) consistently displayed relatively less pupil constriction underwater across both treatment depths compared to *T. e. obscurus*. The greatest pupil constriction recorded was 33.89% of relative pupil diameter for *T. e. obscurus* at the 10 cm depth treatment compared to when in air and the smallest pupil constriction was observed for *T. marcianus* at 91.24% of relative diameter when in air at the 2 cm depth treatment. When running a linear mixed effects model where individual is a random effect, both species (*P* < 0.01) and depth (*P* < 0.001) significantly affect percent of pupil constriction. Between species, there is a 24.3% difference in pupil constriction and between depth, there is an 8.58% difference. Regarding Cohen’s *d* effect sizes, species had a large effect (*d* = −2.65152; 95% CI: lower: −3.336788, upper: −1.966251); whereas depth had a medium effect (*d* = −0.759407; 95% CI: lower: −1.2468517, upper: –0.2719623).

#### Head shape

**Fig. 7:**
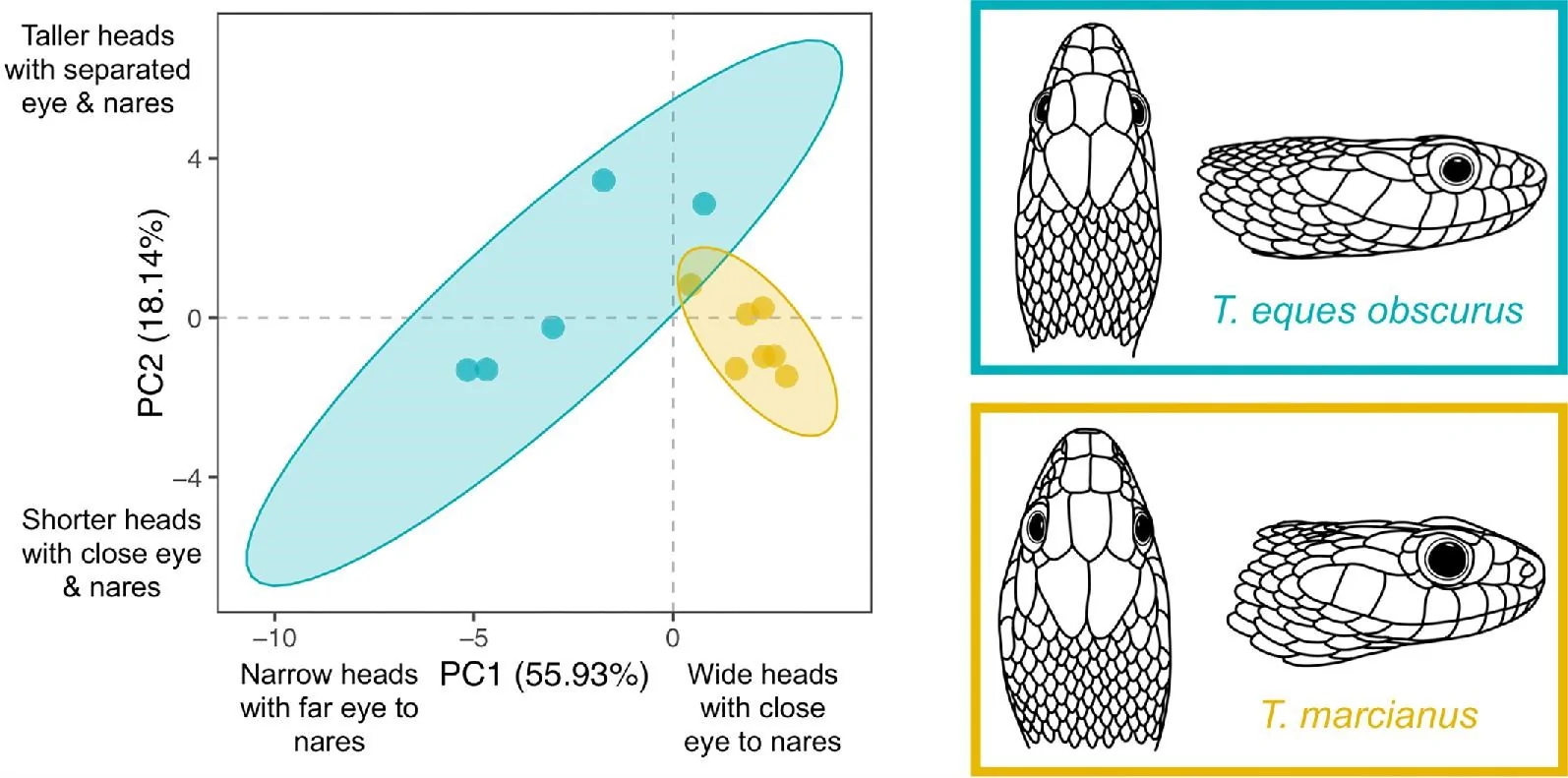
Principal component analysis plot and representative dorsal and lateral drawings of head shape variation in *T. e. obscurus* and *T. marcianus*. PC1 and PC2 explain 74.07% variation in the data set.

In our PCA of the 11 individuals tested, 90% of the variation in head shape was explained by the first 10 principal components (PCs). The first two PC axes together represented >50% of the variation in head shape (PC1: 55.93%; PC2: 18.14%; Fig. 7). Measurements with the highest factor loadings on PCs 1–2 were head widths (HW, HWLSL, HWFSL), head heights (HH, HHP), head lengths (HLRA, HLV), eye to lip (EL), nare to lip (NL), eye to nare (EN), nare to nare (NN), and eye to eye (EE) (Fig. 7; Fig. S1). PC1 (55.93%) represented a majority of the variation in head shape with wider heads and close eye to nares positively skewed and narrower heads with far eye to nares negatively skewed. PC2 (18.14%) accounted for a smaller percent of variation with taller heads and separated eye and nares being positively correlated and shorter heads with close eye and nares being negatively associated.

## Discussion

Our aims were to determine whether naïve captive-bred garter snakes exhibited a chemical prey preference that corresponded to their natural diet and whether the strength of prey preference would correspond to varying degrees of efficiency and accuracy in aquatic prey capture behavior. Our behavioral assay, tongue flicks, and attacks, strongly supported preference for fish in the obligate aquatic forager, *T. e. obscurus. Thamnophis marcianus* showed strong interest in all prey extracts as indicated by elevated tongue flicks. The number of attacks made were highest for trials where swabs were scented with worm and mouse extracts. The strong response to fish by both species supports their reliance on the aquatic environment for feeding.

The interest of *T. marcianus* for worm and mouse extract supports the idea that *T. marcianus* is more of a generalist and shows interest for different prey. Despite the heightened tongue flicks for the control, *T. marcianus* never attacked swabs with distilled water suggesting some ability to differentiate between prey and non-prey extracts.

These behavioral results support recent findings that naïve neonate snakes exhibit preference for their wild prey types (Emerson and Johnson 2024). The fact that naïve, captive-bred snakes exhibit prey preferences for the diet of wild populations is a persistent theme in chemosensory studies (Burghardt 1975, Saviola et al. 2012, Krause et al. 2024,) and enabled us to make predictions about aquatic foraging.

Our results reveal strong prey preference for fish by even naïve, captive bred *T. e. obscurus*. Individuals of *T. e. obscurus* have been reported to only have stomach contents containing different species of fish in their native range (Conant 2003). Therefore, prey preference for fish extract in these captive-bred individuals supports the idea of the obligate aquatic forager specialization described in the literature by wild *T. e. obscurus*. Our chemosensory experiments also show no strong preference toward prey by *T. marcianus*, and support its categorization as a terrestrial-aquatic generalist with fish being slightly preferred making it a facultative aquatic forager along the aquatic dependence continuum (Carrillo and Mehta 2023). As our experiments are among the few chemosensory prey-preference studies performed in these two species (ours is the first for neonate *T. e. obscurus* and the second for neonate *T. marcianus*: Rayburn 1990), our results highlight the predictive power of documented dietary data on prey capture ability even in naïve snakes.

Although *T. marcianus* is considered a terrestrial-aquatic generalist that is thought to prefer the use of lateral strikes, our results show that naïve individuals used forward strikes in deep water. Forward striking is considered an aquatic specialist foraging strategy (Drummond 1983) and is often found with other aquatic specialist traits such as greater visual acuity, and a hydrodynamic head shape. We anticipated *T. marcianus* to prefer lateral striking in shallow water as documented in another generalist species, the common garter snake, *T. sirtalis*. Lateral or sideways sweeping is thought to increase the likelihood of prey capture for snakes with less visual acuity (Alfaro 2002). For example, the fish and amphibian specialist, the Sierra garter snake (*T. couchii*) is able to constrict its pupil to one-third its diameter in air compared to generalists *T. sirtalis* and *T. elegans* who constricted their eyes to about three-fourth times their diameters in air, respectively (Schaeffel and De Queiroz 1990). In this study, we observed that aquatic specialist *T. e. obscurus* was also able to constrict its pupils underwater to 54% of its diameter in the shallow-water treatment and 47% of its diameter in the deep-water treatment. This is in comparison to *T. marcianus* who was recorded constricting its pupils to a much-limited extent (66% of its diameter) in the shallow-water treatment and 62% of its diameter in the deep-water treatment (Fig. 5). Aquatic specialists with greater visual acuity have been shown to strike from further away. Striking from further away should suggest better visual acuity since a snake with better vision and accuracy should be more capable at a distance compared to a less specialized one who may opt to close the gap for greater success. Future work, can look into strike distance in these species. The preferential use of fast forward strikes as opposed to lateral sideways sweeps in deep water by *T. marcianus* was not limited by reduced visual acuity. At fast speeds, the high drag forces from opening the jaws (bow wave) are less pronounced (Van Wassenbergh et al. 2009) despite greater gape angles needed to catch agile fish. The effects of hydrodynamic drag can be further be compounded by head shape where aquatic foraging in *Thamnophis* sp. has converged to be leaner or narrower in the anterior part of the head to reduce drag (Segall et al. 2016). Our geometric morphometrics analysis of head shape revealed that the aquatic specialist *T. e. obscurus* had shorter and narrower heads in comparison to the terrestrial-aquatic generalist *T. marcianus* which can help explain preference for fast forward or lateral sidesweeping strike mode in shallow, wading water. Given that *T. e. obscurus* is a piscivore with fish prey described as having an elongated-shape and *T. marcianus* is more of a generalist with prey described as bulky-shaped, their head shapes may differ based on their diet as observed in other aquatic snakes (Segall et al. 2020). Despite limited visual acuity and a less slender head, the use of forward striking in deep water by *T. marcianus* suggests behavioral convergence for catching prey in deep water. Depth provides a greater three-dimensional space and fast forward striking may be necessary to capture agile prey that may not be repeatedly encountered.

Similarly, *T. marcianus* was able to vary its ability to capture prey in deeper water by using larger gape angles similar to *T. e. obscurus* who also increased its gape angle upon prey capture in the deep-water treatment. *Thamnophis e. obscurus* had the largest mean gape of 46.28° across both depth treatments. Previous work on the Sierra garter snake (*T. couchii*), a facultative aquatic forager (specializing in frogs and fish) was recorded to have a mean maximum gape angle of 39.32° (Alfaro 2002). Similarly, in the same study, the Narrow-headed garter snake (*T. rufipunctatus*), a frog and fish specialist (Gori 2014), averaged 38.81°. The generalist, *T. sirtalis* who feeds on frogs, fish, as well as birds, lizards, rodents, and invertebrates (Heptinstall et al. 2024) averaged 37.70°. And finally in our study, *T. marcianus*, a terrestrial-aquatic forager who according to the literature feeds on leeches, worms, lizards, fish, tadpoles, and frogs, was found to have a mean maximum gape angle of 24.73° across both depth treatments. Our results showcase that gape angles are specialized for the particular aquatic prey and environment in which garter snakes forage. Specifically, we can then expect gape angles to increase with degree of specialization from terrestrial to aquatic or facultative to obligate aquatic foraging. As *T. e. obscurus* is considered the most specialized for the aquatic environment as a piscivore and obligate aquatic forager, it holds the greatest mean gape angle recorded in garter snakes. On the contrary, as *T. marcianus* is considered a facultative aquatic forager, it has the lowest mean gape angle recorded. This demonstrates that the deep-water context is perceived by snakes as requiring different prey capture tactics.

Both the specialist and generalist were able to adapt to the deeper aquatic arena and converge on relatively wider gape angles. The deep-water context, reveals the species’ ability to be behaviorally flexible in a more challenging environment. For example, this increase in gape angle may be due to requiring a larger surface area or “casting a wider net” to capture prey in a larger three-dimensional space. Gape angle kinematics has been shown to vary with predatory successful strikes versus predatory flawed strikes in northern Pacific rattlesnakes (Kardong 1986). Previous work by Alfaro (2002) combined gape angles from successes and fails; however, for our work, we only analyzed successes in order to get a better representation of kinematics of capture success.

Our aquatic experimental foraging trials at two different treatment depths revealed that *T. e. obscurus* was a more accurate forager taking less strikes to successfully capture aquatic prey. Better accuracy may due to factors such as better vision. Aquatic specialist garter snakes, such as the western aquatic garter snake, (*T. couchii*), have been shown to have better visual acuity underwater, such as constrict their pupils to a greater degree, than generalist species (Drummond 1983; Schaeffel and de Queiroz 1990).

We defined efficiency as time it takes to successfully capture prey and found *T. e. obscurus* to be a more efficient forager across the two treatments depths, taking less time to successfully capture prey. Using less time to capture prey allows more time for other behaviors that can increase fitness by reducing the energetic cost associated with locomotion (Norberg 1977), especially underwater on a breath-hold, moving in a medium more viscous than air, and chasing evasive prey (Webb 1984).

Capturing mosquito fish by the head resulted in significantly faster transport times for *T. e. obscurus* compared to the body or tail. However, despite head transport being fastest in terms of time to swallow whole and consequently less time jaw-walking over prey, *T. e. obscurus* did not preferentially do so. In fact, both species captured fish by the head, body and tail with similar proportions. This may be due to chasing evasive prey in a three-dimensional space. For example, there are complexities associated with foraging in the aquatic environment as a breath-hold diver where factors such as vision, chemosensory ability, and experience may play a role in foraging efficiency (number of strikes, time to prey capture).

Moreover, while our analyses only evaluated prey capture successes, there were many instances of a snake capturing the fish by a body part that facilitated escape. For example, capturing fish by the tail often led to a failure since it allowed the fish more lateral movement or thrashing behavior. Retaliatory thrashing behavior by the fish in the deep-water treatment often led to the snake attempting to subdue the fish prey by pinning the fish with its body for more control as it jaw-walked over the prey until fully transported. Snakes also attempted to subdue prey via rotating along the longitudinal axis or “death rolling.” Similarly, an individual *T. e. obscurus* engaged in “knotting” itself during prey processing following a successful prey capture attempt. These behaviors were most often performed in the deep-water treatment. This “death roll” behavior was observed in both species and often resulted in successes perhaps due to disorienting the prey.

While transporting prey underwater at shallow depths was fastest, both species transported prey above and below water. At shallow depths, resurfacing was more common presumably due to the low energetic cost as individuals simply lifted their heads above water.

Finally, in terms of total time spent foraging underwater, both species spent more time foraging in deep water with *T. e. obscurus* spending less time overall, presumably since it was a more accurate forager. For example, when *T. eques obscurus* captured a fish underwater, resurfacing occurred quickly, while *T. marcianus* continued to forage underwater. This continued foraging time allowed *T. marcianus* to capture two fish in a single foraging bout in deep water. The average time spent foraging between intervals in these repetitive successful prey captures without surfacing by *T. marcianus* was 63.3 s (with a maximum of 118 s). As *T. marcianus* was a less accurate forager, it took more time to accomplish the same task and thus individuals spent more time foraging in comparison to *T. e. obscurus*. Either way, sustained foraging in deep water is necessary for capture success and since *T. marcianus* was able to extend its time underwater, this shows that the generalist was able to converge in sustained underwater searching of prey much like the more aquatic snake, *N. sipedon* (Young 1991).

It is important to note that *T. e. obscurus* is one of seven subspecies of Mexican garter snakes (*T. eques*) which independently evolved around the freshwater lakes or its remnants of the transvolvanic belt of Mexico (Conant 2003, Gori 2014). Our species is endemic to Lake Chapala, Mexico’s largest freshwater lake with a maximum depth of 9.8 m (De Buen 1945). Convergence in behavior and morphology for aquatic foraging may subsequently vary in other subspecies that inhabit other lakes with their own unique ecosystems, including endemic prey availability and dynamic water levels that change with precipitation.

In summary, our naïve captive-bred individuals of *T. e. obscurus* preferred the chemical cues of fish while *T. marcianus* had preferences for mouse and worm. These initial experiments allowed us to make predictions on the foraging ability of these same individuals as adults in a naïve context at two water treatment depths: shallow and deep. *Thamnophis e. obscurus* used less strikes and was faster at capturing prey at both depths highlighting its skill as an aquatic specialist. However, *T. marcianus* adopted specialist behaviors in the deep-water context: spending more time underwater, using forward striking, and capturing fish with greater gape angles than in the shallow-water context. Our study revealed that a generalist can be flexible in different environmental contexts but can also converge in specialist foraging tactics when necessary. The results of this work highlight the complexity of aquatic foraging in a three-dimensional space. We observed more consistent and efficient foraging in the aquatic specialist across contexts, but increased water depth influenced how a semi-aquatic generalist modified their foraging strategy to converge in “specialist” adaptations to be successful in deep water.

## Supplementary Material

obag030_Supplemental_File

## Data Availability

All data and R scripts are available on the Stanford Digital Repository as part of the Stanford Data Research Collection and can be accessed via the ORCID iD: https://doi.org/10.25740/rr096wt9885.
